# Human Nanoplatelets as Living Vehicles for Tumor-Targeted Endocytosis In Vitro and Imaging In Vivo

**DOI:** 10.3390/jcm12041592

**Published:** 2023-02-17

**Authors:** Lu Dai, Yehong Liu, Shuang Ding, Xiaowei Wei, Baoan Chen

**Affiliations:** 1 Department of Oncology, Nanjing First Hospital, Nanjing Medical University, Nanjing 210006, China; 2 Department of Hematology, Medical School, Southeast University, Nanjing 210009, China

**Keywords:** engineered nanoplatelets, tumor targeting, in vivo imaging, endocytosis, myeloma xenotransplants

## Abstract

Recent studies have shown human platelets can access the tumor microenvironment by passive diffusion across capillaries or via activated immune cells. In a previous study, we exploited this affinity of platelets for tumor cells as part of a new approach to target tumors with modified platelets. Therefore, the engineering of human nanoplatelets as living vehicles for in vivo tumor-targeted near-infra-red fluorescence (NIRF) imaging and the delivery of cytotoxins to tumor cells by endocytosis are described in this study. Nanoplatelets with an average diameter of 200 nm were prepared by mild sonication of kabiramide C (KabC)-loaded human platelets. The sealed plasma membrane of the nanoplatelets allows them to accumulate and retain membrane-permeable chemicals, such as epidoxorubicin (EPI) and KabC. Tumor-targeted imaging functionalities were engineered on the nanoplatelets by surface-coupling transferrin, Cy5 and Cy7. High-resolution fluorescence imaging and flow cytometry analyses showed that the nanoplatelets loaded with EPI and Cy5 targeted human myeloma cells (RPMI8226 cells) that over-expressed the transferrin receptor. The endocytosis of the nanoplatelets by RPMI8226 cells was transferrin-dependent and induced apoptosis. The test results also showed that the nanoplatelets functionalized with transferrin and Cy7 and injected in mice bearing RPMI8226 cells-derived myeloma xenotransplants accumulated in the tumor tissue and could be used for high-contrast in vivo NIRF imaging of early-stage tumors. Nanoplatelets represent a new class of living nano-vehicles that may efficiently target and deliver therapeutic agents and imaging probes to diseased tissues including tumors.

## 1. Introduction

Platelets are small, anucleate, and discoid-shaped closed-membrane structures that circulate in the vascular system where they play a vital role in hemostasis [1]. A healthy adult has about 4 × 10^12^ platelets, and each day about 10^8^ aged platelets are removed by the liver and spleen, and around 10^8^ mature platelets are developed from cytomegakaryocytes [2]. Although platelets are primarily confined to the vascular system, they may also accumulate in sites of inflammation and tumors [1,2,3,4]. Platelets are believed to access the tumor microenvironment across tumor vessels or via activated immune cells [1,3,4,5]. Recent studies have shown the activation of platelets in the tumor microenvironment promotes angiogenesis and may help metastatic tumor cells evade detection by the immune cells. This phenomenon is called “platelet mimicry” [1,3,4,5]. We have exploited this affinity of platelets for tumor cells to design a new method to produce modified human platelets as targeted carriers for tumor imaging [6]. In particular, we introduced novel tumor-targeting and in vivo imaging functionalities in human platelets by surface-coupling transferrin and internal-loading or surface-coupling of near-infrared fluorescence (NIRF) probes, respectively [6]. Repurposed platelets offer key advantages over artificial nanoparticles for tumor-targeting imaging, including longer circulation times (up to 9 days vs. a few hours), which help to increase the chances of encounter with deep-seated tumor cells, while their large size allows them to transport an internal molecular cargo about 4000-fold greater than that possible using 100 nm nanoparticles. These advantages were recently exploited by our group for in vivo imaging with high specificity of early-stage, intra-cranial myeloma tumors in living mice [6].

We also demonstrated by an in vitro study that repurposed platelets configured with transferrin and Cy5 on their surface targeted specifically RPMI8226 cells, which are known to highly express the transferrin receptor on their surface. Surprisingly, a small population of these repurposed platelets appeared to be internalized [6], which was an unexpected finding, as human platelets with a diameter of over 2 μm should be too large for endocytosis [7]. We speculated at the time that the Cy5-fluorescence stain arose from the endocytosis of small platelet fragments that were generated during the repurposing reactions [6]. In this study, compelling evidence to interpret how human platelet-derived nanoplatelets can be engineered as vehicles for both high-contrast in vivo imaging and their efficient delivery to early-stage tumors is provided. Molecularly functionalized human nanoplatelets represent a new class of vehicles for tumor imaging and tumor therapy. Functional nanoplatelets are very different in their design, make-up, and functional properties from the recently described nanoparticles cloaked with denatured fragments of platelet membranes [8,9,10,11].Tumor-targeting nanoplatelets offer distinct advantages compared to hybrid nanomaterials in tumor-targeted delivery, which is similar to that of their mother intact platelets and leading to a privileged access to the tumor microenvironment, much longer circulation times that increase their chance of encountering deep-seated tumor cells, and clearance by the liver and spleen. Moreover, nanoplatelets were shown to accumulate membrane-permeable chemicals, such as epidoxorubicin (EPI) and kabiramide C (KabC). This finding also highlights additional and unique features, indicating that the plasma membrane of nanoplatelets is sealed, functional, and right-side-out, as it retains its internal contents against the effects of passive diffusion. These features were exploited for the design of tumor-targeting nanoplatelets as living vehicles that can transport therapeutics and NIRF probes to tumors in immuno-compromised mice for high-contrast imaging and to induce apoptosis in myeloma xenotransplants in the future.

## 2. Materials and Methods

### 2.1. Materials

RPMI8226 cells (multiple myeloma cells) were purchased from the cell bank of the Chinese Academy of Sciences. Human platelets were obtained from the Jiangsu province blood center. The platelets were cultured for 5 days in vitro and resuspended in citrate saline (0.006 M tri-sodium citrate/0.154 M NaCl, pH 6.8) with 5% bovine serum albumin at 3–5 × 10^8^ platelets/mL. After centrifugation at 2500 rpm for 10 min, the sedimented platelets were resuspended in modified Hanks’ buffered salt solution (mHBSS; 0.17 M NaCl/6.7 mM KCl/1.0 mM MgSO_4_/0.5 mM K_2_HPO_4_/2.8 mM Na_2_HPO_4_/13.8 mM dextrose, pH to 7.2 with 1.4% NaHCO_3_) for in vitro studies or in normal saline for in vivo studies. KabC was isolated as previously reported [12,13,14]. Human transferrin, epidoxorubicin (EPI), maleimide-benzoic acid succinimide ester (MBS), phenyldimaleimide, maleimide-benzoic acid succinimide ester (PDM) and Traut’s reagent (iminothiolane) were purchased from Sigma-Aldrich LLC. (Saint Louis, MO, USA). Cy5-NHS and Cy7-NHS were purchased from GE Healthcare (Pittsburgh, PA, USA).

### 2.2. Transferrin Conjugates

PDM-transferrin was produced according to our recent publication [6]. A transferrin conjugate (transferrin-Cy5-MBS) was produced in phosphate-buffered solution (PBS) by incubating transferrin (5 mg/mL) with Cy5-NHS (0.2 mg/mL) and MBS (0.1 mg/mL). The reactive molecules were added to dimethyl formamide (DMF) stock solutions. The unbound probes were removed from the transferrin conjugates using PD-10 chromatography. The eluted transferrin conjugates were characterized in our previous study [6]. The MBS/Cy7-conjugate of transferrin was produced in the same way as that described above. All the transferrin conjugates were stored at −20 °C in 100 μL aliquots.

### 2.3. Nanoplatelets

Freshly isolated human platelets loaded with KabC were sonicated using a VibraCell ultrasonicator (VCX130PB 130 W 20 kHz; Sonics & Materials, Inc., Newtown, CT, USA). Forward scatter (FSC) vs. side scatter (SSC) plots from flow cytometric analyses of platelet homogenates were recorded after 15 min of sonication in a single 2 mm probe in mHBSS on ice at 20% amplitude, with 20 s on, 10 s off pulses. Reference latex beads (200 nm) were used as a standard to distinguish intact platelets from nanoplatelets.

### 2.4. High-Performance Liquid Chromatography (HPLC) and Cell Apoptosis Assay

RPMI 8226 cells (2 × 10^5^ cells/well) were seeded into wells in 12-well plates and allowed to grow overnight. EPI was loaded in nanoplatelets with and without surface-coupled transferrin for 1 h at 5 mg/mL, 10 mg/mL and 15 mg/mL. After a 1 h of treatment, the nanoplatelets were isolated from free EPI by washing and centrifugation at 12,000 rpm for 30 min. Then, the EPI-loaded sedimented nanoplatelets were placed in tubes on dry ice, 4 mL of 80% HPLC-grade methanol (cooled to −80 °C) was added, and incubation was carried on for 20 min. After centrifuging the lysate/methanol mixture at 12,000 rpm for 5 min at 4 °C, the metabolite-containing supernatant was transferred to a new tube on dry ice. Then, 500 μL of 80% methanol (−80 °C) was added to the pellet in each tube, and then the tubes were oscillated for 1 min at 4 °C. After spinning the tubes at 12,000 rpm for 5 min at 4 °C, the supernatants were transferred to new tubes on dry ice and then SpeedVac/lyophilized to dryness. The dried-metabolite samples were stored at −80 °C for further analysis. HPLC was used to quantify the final EPI concentration of the EPI-loaded nanoplatelets preparation. We treated 1 mL of RPMI 8226 cells suspensions in 12-well plates with 200 μL of the EPI-loaded nanoplatelets preparation or with free EPI (0.1 mg/mL) for 4 h. Then, the cells were centrifuged to remove the nanoplatelets or the free EPI, and further cultured for 24 h in fresh medium. These cells were analyzed for apoptosis using the APC-Annexin V and DAPI apoptosis kit (BD Company, Franklin Lakes, NJ, USA) and flow cytometry.

### 2.5. Confocal Fluorescence Microscopy

Visible fluorescent probes were either loaded in the cytoplasm or coupled to the surface of the nanoplatelets. Fluorescence images were taken by a Zeiss 700 microscope after selective excitation of DAPI and CY5 at 405 nm and 639 nm, respectively. Fluorescence emission was recorded over an appropriate wavelength range for each of these probes. Images of samples containing mixtures of functionalized nanoplatelets and RPMI8226 cells in vitro were acquired.

### 2.6. Flow Cytometry

The labeled populations of nanoplatelets with Cy5-transferrin on the surface or loaded with EPI in the cytoplasm were quantified by a Becton and Dickenson FACS Calibur flow cytometer. We recorded 10,000 events in each sample using the fluorescence of EPI or Cy5 through the FL2 or the FL4 channel, respectively. These data were analyzed using the FlowJo V3.2 software suite (Tree Star, Inc., Stanford, CA, USA). For the analysis of the nanoplatelets’ size, the events were gated based on size (forward scatter, FSC) and granularity (side scatter, SSC). The FSC vs. SSC plots of platelet sonicates showed that populations with low FSC and SSC reflected the nanoplatelets, while populations with high FSC and SSC reflected intact platelets. Non-fluorescent beads with a 200 nm peak diameter and a diameter range of 150–250 nm (Becton-Dickenson Company, Franklin Lakes, NJ, USA) were used as a size standard and size threshold to distinguish intact platelets from nanoplatelets.

### 2.7. Electron Microscopy

Platelets and nanoplatelets were imaged using a Hitachi 450 SEM (Tokyo, Japan) and a FEI Tecnai G20 Spirit Bio TWIN TEM (Hillsboro, OR, USA) based on an established protocol [15]. The nanoplatelets were first resuspended by pipetting and immediately fixed in an equal volume of 0.1% glutaraldehyde. After 15 min, the fixed nanoplatelets were centrifuged at 12,000 rpm for 30 min and resuspended in 3% glutaraldehyde. The nanoplatelets were adhered to poly-lysine coated glass slides. The glass slides were rinsed and dried for EM imaging.

### 2.8. Mice

Anesthetics were used to minimize pain to animals during the in vivo experiments. Sub-cutaneous and intra-cranial injections of RPMI8226 cells were conducted as described by Dai et al. [6]. The nanoplatelets were suspended in normal saline and immediately injected intravenously in the tail vein (10 μL/g of the body weight). The NOD/SCID mice were euthanatized after intra-peritoneal injection with a 0.016 mL/g solution of 2.5% avertin or CO_2_ anesthesia. All animal experiments were conducted according to protocols of the Medical School of Southeast University.

### 2.9. NIR Fluorescence Imaging

RPMI8226 cells were injected sub-cutaneously or intra-cranially in NOD/SCID mice. NIRF imaging of live mice was conducted by a Caliper IVIS Spectrum Imaging System (Long Island, NY, USA). NIR fluorescence images were recorded using IVIS software. Any fur in the region of the tumor was shaved before in vivo imaging.

## 3. Results

### 3.1. Preparation and Characterization of the Nanoplatelets

**Figure 1:**
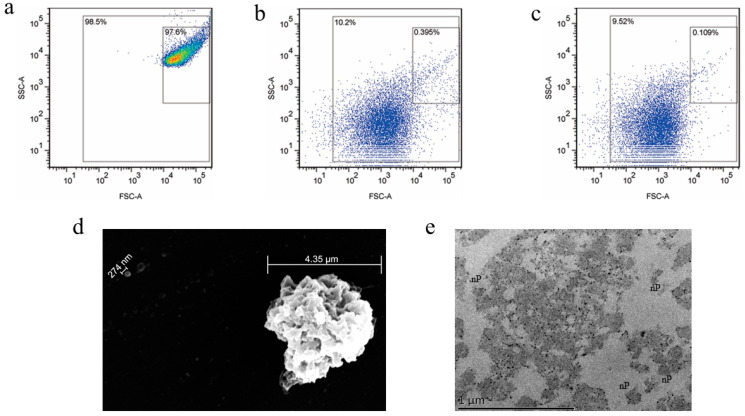
Flow cytometry and electron microscopy (EM) analysis of human platelets and nanoplatelets. (**a**) Flow cytometry analysis of intact KabC-platelets without sonication, (**b**) after sonication at 20% amplitude for 15 min, (**c**) size distribution of reference 200 nm (150–250 nm) beads. Scanning EM (**d**) and transmission EM images (**e**) of KabC-platelets after sonication for 15 min at 20% amplitude.

The nanoplatelets were prepared in high yield by mild sonication of intact KabC-loaded human platelets [6] using a VibraCell ultrasonicator. Flow cytometry was used to optimize the conditions for their sonication-mediated production. The flow cytometry plots in Figure 1a–c shows the final conditions used to prepare the nanoplatelets. FSC indicates the volume size of the particles being measured, and SSC indicates the internal structural property of the particles. In the plots, the lower left corner region with low FSC and low SSC represents the smaller nanoplatelets (Figure 1b), and the upper right corner region with high FSC and high SSC represents the larger intact platelets (Figure 1a). In particular, the nanoplatelets were prepared in high yield by exposing a suspension of KabC-loaded human platelets in mHBSS (3–5 × 10^8^ platelets/mL) on ice to cycles of ultrasound delivered by a VibraCell ultrasonicator at a power of 130 W, 20 kHz for 15 min, with each cycle composed of an on-period at 20% amplitude for 20 s, followed by an off-period of 10 s. The size distribution of these nanoplatelets was similar to that recorded for a suspension of 200 nm reference beads (Figure 1c). The SEM image in Figure 1d shows a pre-fragmented mother platelet with bulb-like, nanoscale surface projections. The TEM image of a platelet at a more advanced stage of sonication in Figure 1e shows a central “mother” platelet, surrounded by closed-membrane nanoplatelets (indicated by “nP”) of 100–300 nm diameter, with a similar internal granular structure as that of the mother platelet. The nanoplatelets in this field of view have a range of shapes that includes non-spherical structures that could be formed from sonication-mediated fragmentation of the mother platelet.

### 3.2. Preparation and Properties of the Functionalized Nanoplatelets

**Figure 2:**
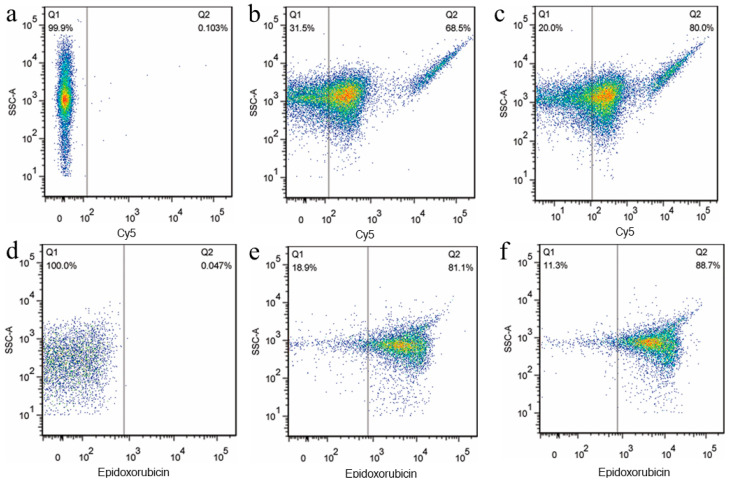
Nanoplatelets surface-coupled with Cy5-Transferrin and cytoplasm-loaded with EPI. (**a**) control nanoplatelets without surface-coupled Cy5-Transferrin, (**b**) nanoplatelets surface-coupled with Cy5-Transferrin at an incubation concentration of 3 µM, (**c**) nanoplatelets surface-coupled with Cy5-Transferrin at an incubation concentration of 9 µM, (**d**) control nanoplatelets without EPI, (**e**) nanoplatelets incubated with EPI at an incubation concentration of 5 mg/mL before adding thrombin (1 U/mL), (**f**) nanoplatelets incubated with EPI at an incubation concentration of 5 mg/mL after adding thrombin (1 U/mL). Nanoplatelets treated with thrombin retained their sequestered EPI, which suggests the plasma membrane was not compromised on activation.

Freshly prepared nanoplatelets (Section 3.1) were resuspended in mHBSS and re-incubated immediately with 5 M KabC for 30 min to reduce their transient self-aggregation. KabC-loaded nanoplatelets were further manipulated to impart novel molecular functionalities for tumor targeting, high-contrast in vitro and in vivo imaging, and the delivery of internalized cytotoxins to tumor cells. The nanoplatelets were equipped with a novel tumor-targeting function by bioconjugating transferrin (Cy5-transferrin) (Figure 2b,c) [6]. A means to detect or to image tumor-bound nanoplatelets in vivo was realized by coupling Cy7-transferrin to their outer surface using the same method. Flow cytometry analysis of the intrinsic fluorescence of EPI showed nanoplatelets accumulated EPI in their cavity during a 60 min incubation period (Figure 2e,f). Nanoplatelets treated with thrombin retained their sequestered EPI (Figure 2f). Collectively, these studies demonstrated that the plasma membrane of the nanoplatelets was not only right side out, but harbored functional esterases on its cytoplasmic face. Moreover, nanoplatelets have the capacity to actively control the transport of small molecules across the plasma membrane [16]. To reduce the impact of aggregation, the nanoplatelets were routinely pipetted before being mixed with cells or injected into a mouse. The protocols used for surface-coupling and loading appear in the Methods section of this study or in our earlier publication [6].

### 3.3. Imaging Interactions between Functionalized Nanoplatelets and RPMI8226 Cells

**Figure 3:**
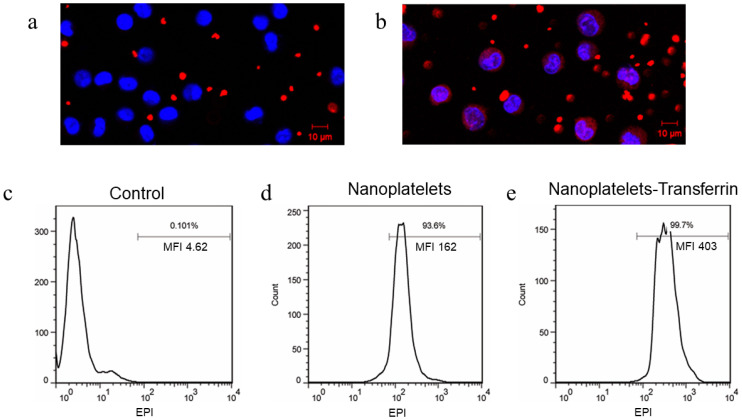
Endocytosis in vitro of functionalized nanoplatelets. Confocal (Cy5/DAPI) overlay image showing nanoplatelet (Cy5) interactions with RPMI8226 cells (DAPI): (**a**) control nanoplatelets surface-coupled only with Cy5; (**b**) test nanoplatelets surface-coupled with transferrin and Cy5. Scale bar, 10 µm. FACS of RPMI8226 cell internalization of nanoplatelets surface-coupled with (**e**) or without (**d**) transferrin and cytoplasm-loaded with EPI. (**c**) nanoplatelets without EPI in cytoplasm were set as blank control. The intrinsic fluorescence of EPI was used for FACS detection in these samples.

Nanoplatelets configured with surface-coupled transferrin and Cy5 and loaded with KabC were shown by fluorescence confocal microscopy to interact specifically with RPMI8226 cells (Figure 3b). In particular, the intensity of Cy5-fluorescence in the cytoplasm of RPMI8226 cells was found to be much stronger for nanoplatelets surface-coupled with transferrin (test) compared to control nanoplatelets without transferrin (Figure 3b, a respectively). One can also infer from the punctate Cy5 staining of RPMI8226 cells and from the transferrin-dependent uptake of nanoplatelets that their internalization in RPMI8226 cells proceeded via receptor-mediated endocytosis. This mechanism is consistent with studies that showed that RPMI8226 cells overexpress the transferrin receptor [6,17]. The robust endocytosis of the nanoplatelets coupled with transferrin was quite different from the behavior we noted for intact platelets coupled with transferrin [6]. In that study, the intact platelets did not show any evidence of being endocytosed, while capable of binding in large numbers to the surface of RPMI8226 cells, presumably because their diameter exceeded the size limit for endocytosis [6,7]. Next, the endocytosis of functionalized nanoplatelets by RPMI8226 cells was explored in more detail. The mechanism by which the functionalized nanoplatelets were uptaken by RPMI8226 cells was investigated by FACS taking advantage of EPI fluorescence within nanoplatelets. The mean intensity of EPI fluorescence in RPMI8226 cells increased 2.49-fold following the addition of nanoplatelets surface-coupled with transferrin (Figure 3e), compared to that measured for nanoplatelets without transferrin (Figure 3d).

### 3.4. Apoptosis of RPMI8226 Cells Triggered by the Endocytosis of the Functionalized Nanoplatelets

**Figure 4:**
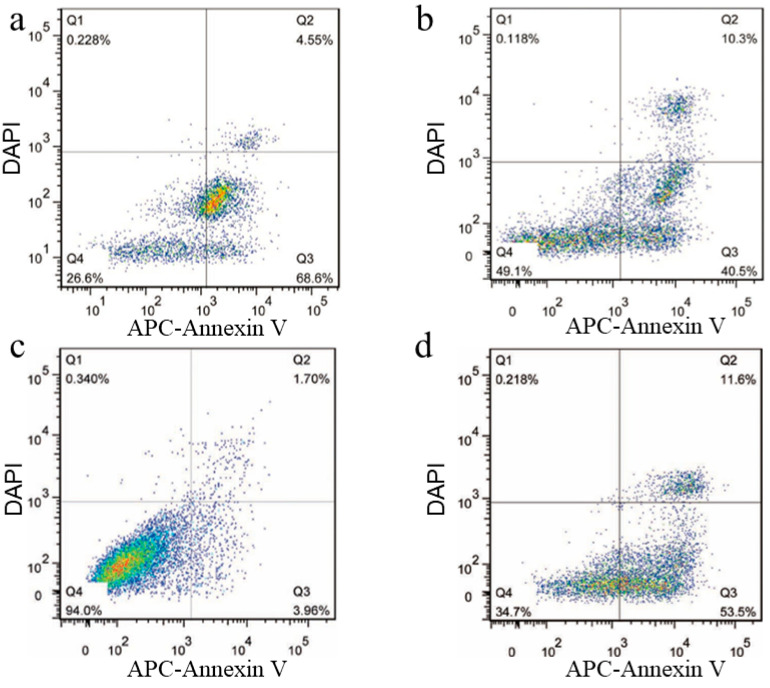
Quantification of early apoptosis in RPMI8226 cells using a standard FACS-based apoptosis assay that measures the labeling of RPMI8226 cells with APC-Annexin V and DAPI. The data are expressed in percentages of apoptotic cells—the results are representative of two independent experiments. (**a**) RPMI8226 cells treated with transferrin-coupled nanoplatelets loaded with EPI (incubation concentration of 5 mg/mL); (**b**) RPMI8226 cells treated with nanoplatelets without transferrin but loaded with EPI (incubation concentration of 5 mg/mL); (**c**) control RPMI8226 cells without nanoplatelet treatment; (**d**) RPMI8226 cells treated with free EPI at 0.1 mg/mL. Cells were exposed to the treatments for 4 h, followed by sufficient washing to remove the nanoplatelets; the cells were further cultured 24 h. In each FACS scatter diagram, Q2 (right upper) represents the population of late-apoptosis cells, Q3 (right lower) represents the population of early-apoptosis cells; Q4 (left lower) represents the population of live cells. The experiments were repeated three times. These studies showed that the nanoplatelets with transferrin and EPI had stronger a apoptosis-inducing effect than control EPI-loaded nanoplatelets without transferrin.

Flow cytometry was also used to show that the internalization of the functionalized nanoplatelets triggered EPI-induced cell death. In these studies, RPMI8226 cells were treated for 4 h with EPI-loaded nanoplatelets with or without surface-coupled transferrin, followed by 24 h at 37 °C in fresh culture medium. The percentage of RPMI8226 cells that survived (Q4) the treatment with EPI-loaded nanoplatelets coupled with transferrin was significantly lower, at 26.6% (Figure 4a), compared to that of RPMI8226 cells treated with EPI-loaded nanoplatelets without transferrin (49.1%; Figure 4b). Also significant was the finding that far more RPMI8226 cells treated with EPI-loaded nanoplatelets coupled with transferrin entered early apoptosis (Q3, 68.6%, Figure 4a), compared to cells treated with EPI-loaded nanoplatelets without transferrin (40.5%; Figure 4b). Separately, a control study showed 94% of RPMI8226 cells survived a treatment with saline, with only 3.96% of cells entering early apoptosis (Figure 4c). In comparison 34.7% of RPMI8226 cells were shown to survive the exposure to free EPI (0.1 mg/mL), with 53.5% of these cells entering early apoptosis (Figure 4d). HPLC was used to quantify the final EPI-loaded nanoplatelet concentration, which resulted to be approximately 0.84 ± 0.13 mg/mL, 1.20 ± 0.25 mg/mL and 1.46 ± 0.41 mg/mL after incubating the nanoplatelets with EPI, respectively, at 5 mg/mL, 10 mg/mL and 15 mg/mL. In the cell apoptosis assay, 200 μL of the EPI-loaded nanoplatelets preparation at an EPI-loaded concentration of approximately 0.84 mg/mL was added to 1 mL of RPMI 8226 cell suspension. The final EPI reaction concentration of the EPI-loaded nanoplatelets with RPMI 8226 cells in a 12-well plate was approximately 0.14 mg/mL, as shown in Figure 4a,b. These studies showed that transferrin-coupled nanoplatelets loaded with EPI were more effective at inducing RPMI8226 apoptosis compared to EPI-loaded nanoplatelets without transferrin or free EPI. Next, we explored the diagnostic (in vivo imaging) and therapeutic potential of EPI-loaded nanoplatelets coupled with transferrin and Cy7 by analyzing their interactions with RPMI8226 cells within myeloma xenotransplants in immuno-compromised mice.

### 3.5. In Vivo Imaging of Functionalized Nanoplatelets in Myeloma Xenotransplants

**Figure 5:**
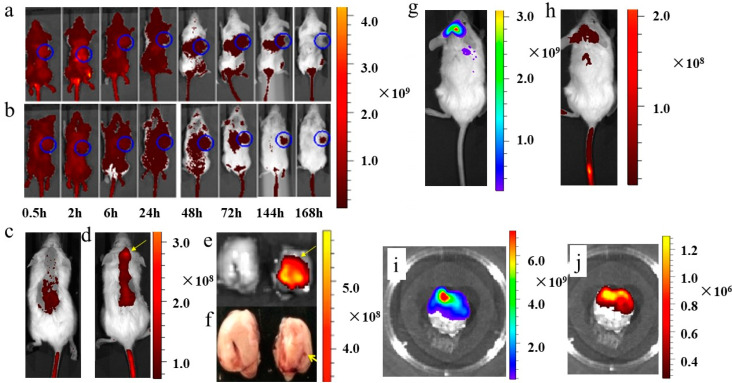
In vivo imaging of nanoplatelets in mice bearing sub-cutaneous and intracranial multiple myeloma xenotransplants. (**a**) Representative time-course images of the distribution of control Cy7-coupled nanoplatelets (without transferrin) injected on day 15 in a mouse bearing a sub-cutaneous tumor; the experiments were repeated two times. (**b**) Representative time-course images of the distribution of test Cy7- and transferrin-coupled nanoplatelets injected on day 15 in a mouse bearing a sub-cutaneous tumor; the experiments were repeated two times. (**c**) Images of a representative mouse bearing an intra-cranial tumor, 24 h after injecting the control Cy7-coupled nanoplatelets (without transferrin); the experiments were repeated three times. (**d**) Images of a representative mouse bearing an intra-cranial tumor 24 h after injecting the nanoplatelets coupled with Cy7 and transferrin; the experiments were repeated three times. (**e**) Cy7-fluorescence image of an excised brain sliced into the left and right lobes to show that the fluorescence signal was restricted to the right lobe where the RPMI8226 cells were injected. (**f**) I-phone-acquired photograph of the excised sliced brain of the mouse shown in (**d**,**e**) showing the site of tumor cells injection in the cranium. (**g**) Image of a mouse bearing an intracranial xenotransplant composed of GFP-transfected RPMI8226 cells. (**h**) Cy7-fluorescence images of the same mouse in Figure 5g. (**i**) GFP-fluorescence image of the excised brain from the same mouse shown in Figure 5g,h. (**j**) Cy7-fluorescence image of the same excised brain shown in Figure 5i. The lookup tables presented report units of radiant efficiency (photons·sec^−1^·cm^−2^·steradian^−1^)/μW·cm^2^.

Sub-cutaneous and intra-cranial myeloma xenotransplants were shown to develop after injecting RPMI8226 cells in NOD/SCID mice. In one set of studies, mice bearing sub-cutaneous myeloma xenotransplants (15 days post injection) were injected with a suspension of nanoplatelets functionalized with Cy7, loaded with EPI and coupled to transferrin (test; n = 3); the control nanoplatelets in this experiment lacked transferrin (10 μL of nanoplatelets per gram of body weight). At given times, indicated in the legend of Figure 5a,b, Cy7-emission in anaesthetized mice bearing a sub-cutaneous myeloma xenotransplant (indicated by a blue circle in Figure 5a,b) was imaged using a Caliper NIRF spectral imaging instrument. Overlay images of Cy7 fluorescence and reflected white-light were generated for each mouse, with representative images being shown for the control (Figure 5a) and test nanoplatelets (Figure 5b). While the intensity of Cy7-fluorescence on the dorsal side of each mouse was high and uniform during the first 24 h from the injection, it decreased rapidly thereafter and, after 48 h, was largely absent from the cranium (Figure 5a,b). After 168 h, the intensity of Cy7 fluorescence from the control and test nanoplatelets was very low over the entire dorsal surface (Figure 5a,b). The time-dependent loss of Cy7 fluorescence from the dorsal side of the mouse was mirrored by an increase of fluorescence in the liver and spleen, both of which are known to clear aged platelets from the circulation [2]. A different pattern of Cy7 staining was found in the sub-cutaneous myeloma xenotransplant in the mouse injected with the test nanoplatelets. The sub-cutaneous tumor emerged from Cy7 fluorescence images at 72 h, which was primarily a consequence of an improvement in signal contrast resulting from the clearance of unbound nanoplatelets from the dorsal region (Figure 5b). Cy7 emission reached a maximum intensity after 72 h and while it decreased to some extent beyond that time, the contrast of the Cy7-labeled tumor remained high for 168 h, presumably because there were far fewer unbound nanoplatelets. These time-series studies highlighted three important features of the prepared nanoplatelets for tumor targeting. First, the nanoplatelets circulated for at least 72 h after injection, during which time they could gain access to deep-seated cells in the tumors’ microenvironment. Second, the long-lived association of the nanoplatelets with targeted tumor cells could be exploited for the delivery of slow-acting therapeutic agents to tumor cells. Third, the nanoplatelets were removed exclusively by the liver and spleen, and the transferrin-coupled nanoplatelets had a stronger tumor-targeting effect than the control nanoplatelets without transferrin (Supplementary Figure S1).

Next, we recorded images of Cy7 fluorescence 24 h after injecting the control nanoplatelets without transferrin (Figure 5c) and the test nanoplatelets surface-coupled with transferrin (Figure 5d) in mice, injected 5 days before on the right side of the intra-cranial cavity with RPMI8226 cells for (Figure 5c–f). Representative images of these studies showed very little Cy7 signal from the cranium of the control mouse (Figure 5c), whereas Cy7 emission was found concentrated in an area in the cranium of the test mouse (indicated by a yellow arrow in Figure 5d). This finding was confirmed by recording Cy7 fluorescence and white light images of the excised brain from the test mouse, as shown in Figure 5d, and its divided hemispheres (Figure 5e,f). This image shows that the Cy7 signal originated exclusively from the right hemisphere of the brain (the site of the RPMI8226 cell injection), which is highlighted in the photographic image of the same sliced brain shown in Figure 5f.

In a further demonstration of the tumor-targeting and in vivo imaging capabilities of the functionalized nanoplatelets, we imaged Cy7 emission from nanoplatelets injected in mice bearing myeloma xenotransplants derived from intra-cranial transplants of RPMI8226 cells transfected with GFP. In particular, GFP fluorescence was used to image the location of RPMI8226 cells in the myeloma xenotransplant (Figure 5g), while the Cy7 signal was used to image the distribution of the functionalized nanoplatelets 48 h after they had been injected in living mice (Figure 5h) and 15 days after the transplantation of GFP-transfected RPMI8226 cells. The GFP and Cy7 emission signals recorded in vivo showed an extensive overlap around the cranium (Figure 5g,h), a finding that was made clearer by inspections of GFP and Cy7fluorescence images of the excised brain, both of which exhibited similar intensity distributions (Figure 5i,j respectively).

## 4. Conclusions

Human nanoplatelets as living vehicles for imaging myeloma xenotransplants in mice and for the targeted delivery of apoptotic drugs to RPMI8226 cells by endocytosis in vitro were developed in this study. Human nanoplatelets were engineered in vitro with molecular functionalities for tumor targeting, imaging and therapy by loading them with cytotoxins and vital stains and by attaching transferrin to their outer membrane. RPMI8226 cells were shown to internalize the functionalized nanoplatelets via transferrin-mediated endocytosis, which was followed by EPI-induced apoptosis of RPMI8226 cells. After being injected into mice bearing RPMI8226 cell-derived myeloma xenotransplants, the nanoplatelets with transferrin–Cy7 accumulated in the sub-cutaneous and intra-cranial myeloma xenotransplants, where they generated intense NIRF signals that allowed performing high-contrast imaging. These experiments indicate that the functionalized nanoplatelets are effective living vehicles for targeted imaging and the delivery of apoptotic drugs to tumors.

Functionalized human nanoplatelets represent a new class of vehicles for tumor imaging and therapy. They differ in very important ways from the recently described nanoparticles that integrate denatured fragments of platelet membranes [8,9,10,11]. For example, they are present for up to a week in the circulation and associate with their target cells over a similar period and have a closed and functional plasma membrane that shares similarities with that of intact human platelets. In addition, nanoplatelets retain their intracellular cargo of toxins and probes for up to a week without significant leakage. These features allow nanoplatelets to transport their cargo for far longer periods in the circulation compared to hybrid nanoparticles, which should increase their chances of interacting with deep-seated tumor cells. Moreover, as with their mother platelets, nanoplatelets are removed exclusively by the liver and spleen and do not show evidence of off-target binding. Finally, nanoplatelets loaded with EPI and coupled with transferrin were taken up by RPMI8226 cells in vitro where they triggered apoptosis. These findings highlight the potential of cytotoxin-loaded nanoplatelets coupled with special antibodies as a new class of targeted nanoparticles suitable for high-contrast imaging of early-stage tumors and the efficient targeted delivery of therapeutic agents to deep-seated tumor cells.

## Data Availability

All analyzed data within this study can be obtained from the corresponding author on request.

## Supplementary Materials

The following supporting information can be downloaded at: https://www.mdpi.com/article/10.3390/jcm12041592/s1, Figure S1: The kinetics of nanoplatelets distribution upon injection into mice bearing sub-cutaneous RPMI8226 tumors.
