# Supplementary material for: Human Nanoplatelets as Living Vehicles for Tumor-Targeted Endocytosis In Vitro and Imaging In Vivo
Source: J Clin Med. 2023 Feb 17;12(4):1592. doi: 10.3390/jcm12041592 (PMC9966157; doi:10.3390/jcm12041592)
Supplement: Supplementary file 1 [file jcm-12-01592-s001.zip › jcm-2095689-supplementary.pdf]

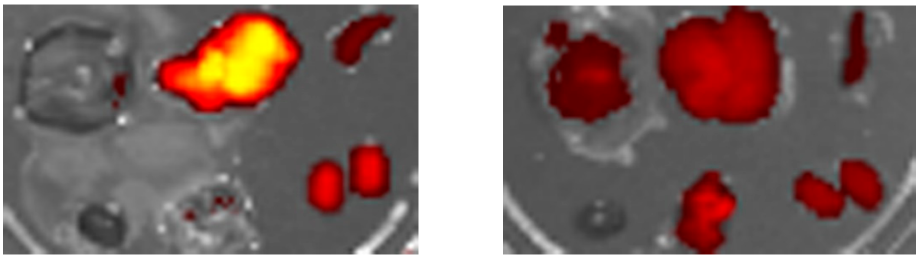

Supplementary Figure S1. The kinetics of nanoplatelets distribution upon injection into mice bearing sub-cutaneous RPMI8226 tumors. Cy7-fluorescence images of the main organs in mice injected with control Cy7-coupled nanoplatelets without transferrin (**left**), as in Figure 5a, or Cy7- and transferrin-coupled nanoplatelets (**right**), as in Figure 5b. From left to right, the organs above are tumor, liver and spleen, respectively, and the organs below are heart, lung and kidney, respectively.
